# Direct Analysis of Mitochondrial Damage Caused by Misfolded/Destabilized Proteins

**DOI:** 10.3390/ijms23179881

**Published:** 2022-08-31

**Authors:** Jannatul Aklima, Sawaros Onchaiya, Tomonori Saotome, Punitha Velmurugan, Taihei Motoichi, Jannatul Naima, Yutaka Kuroda, Yoshihiro Ohta

**Affiliations:** 1 Division of Biotechnology and Life Sciences, Tokyo University of Agriculture and Technology, 2-24-16 Nakacho, Koganei, Tokyo 184-8588, Japan; 2 Department of Biochemistry and Molecular Biology, University of Chittagong, Chittagong 4331, Bangladesh; 3 Department of Bioengineering, Nagaoka University of Technology, Niigata 940-2188, Japan

**Keywords:** dengue envelope protein domain 3, membrane, mitochondria, misfolded protein, thermal instability

## Abstract

Protein quality control is essential for cellular homeostasis. In this study, we examined the effect of improperly folded proteins that do not form amyloid fibrils on mitochondria, which play important roles in ATP production and cell death. First, we prepared domain 3 of the dengue envelope protein in wild type and four mutants with widely different biophysical properties in misfolded/aggregated or destabilized states. The effects of the different proteins were detected using fluorescence microscopy and Western blotting, which revealed that three of the five proteins disrupted both inner and outer membrane integrity, while the other two proteins, including the wild type, did not. Next, we examined the common characteristics of the proteins that displayed toxicity against mitochondria by measuring oligomer size, molten globule-like properties, and thermal stability. The common feature of all three toxic proteins was thermal instability. Therefore, our data strongly suggest that thermally unstable proteins generated in the cytosol can cause cellular damage by coming into direct contact with mitochondria. More importantly, we revealed that this damage is not amyloid-specific.

## 1. Introduction

Quality control of proteins is essential for cell viability, and nascent polypeptides undergo complex, multi-step folding processes before they can become functionally active proteins. Cells possess a variety of quality control mechanisms to deal with improperly folded proteins, and they repair or digest misfolded proteins. However, deficient quality control systems can lead to the accumulation of misfolded or partially folded proteins that can trigger neurodegenerative diseases by interacting with various cellular components [1,2,3,4,5,6,7].

Mitochondria are important cellular organelles that synthesize adenosine triphosphate (ATP) and regulate various cellular metabolism like Ca^2+^ homeostasis, hormone signaling, and cell death under normal conditions. Consequently, mitochondria are involved in the pathological mechanisms of many diseases, as evidenced by the disruption of mitochondrial function, morphology, and dynamics. Amyloid fibrils are a well-known cause of neurodegenerative disease [8,9,10] which can induce mitochondrial damage, leading to serious cellular dysfunction. Although other types of misfolded proteins, such as small oligomeric or monomeric sheets [11] or amorphous aggregates [12,13], have also been observed to cause cellular dysfunction, the specific underlying mechanisms remain unclear.

In this study, we investigated the mechanisms of mitochondrial damage caused by non-amyloid-misfolded proteins. Importantly, we used highly active isolated mitochondria to observe the direct action of added proteins on mitochondria. As a model protein for interaction with mitochondria, we used the domain 3 of the envelope protein (ED3) of dengue virus serotype 3 (DENV3), which is a small β-sheet protein of 106 residues that contains an Ig fold [14] and folds spontaneously. The variants were chosen because a single or a few mutations induced a significant change in the oligomerization state/thermal stability of the protein. We prepared a wild-type DENV3-ED3 and generated four variants with very similar sequences but substantially different biophysical properties, and used them to analyze how biophysical properties of a protein can affect mitochondrial toxicity.

In particular, our study aims to examine whether a protein (any protein) becomes toxic upon aggregation/ destabilization. We chose DENV3-ED3 as a model protein for a practical reason: Several variants with single or few mutations that exhibited uniquely different biophysical properties in terms of oligomerization and thermal stability [15,16,17] were available, and DENV3-ED3′s native structure was well characterized [18]. Altogether, this study provided insights into the mechanisms of cellular and in particular mitochondrial damage caused by non-amyloid-misfolded proteins.

## 2. Results

### 2.1. Effects of DENV3-ED3 Variants on Mitochondrial Membrane Potential

Mitochondrial membrane potential is an important indicator of mitochondrial activity as it affects the uptake of cations into mitochondria. Therefore, we first evaluated the effects of the DENV3-ED3 variants’ physico-chemical properties on mitochondrial membrane potential using mitochondria isolated from porcine hearts or HeLa cells. The variants were chosen from a larger pool of mutants, and named according to the number and type of amino acids added to the C-terminus of the protein [19], as summarized in Table 1 and Figure S1. The structural/physical origin of the aggregation/destabilization has been reported in our previous studies and mainly originates from hydrophobic effect and atomic clashes.

**Figure 1:**
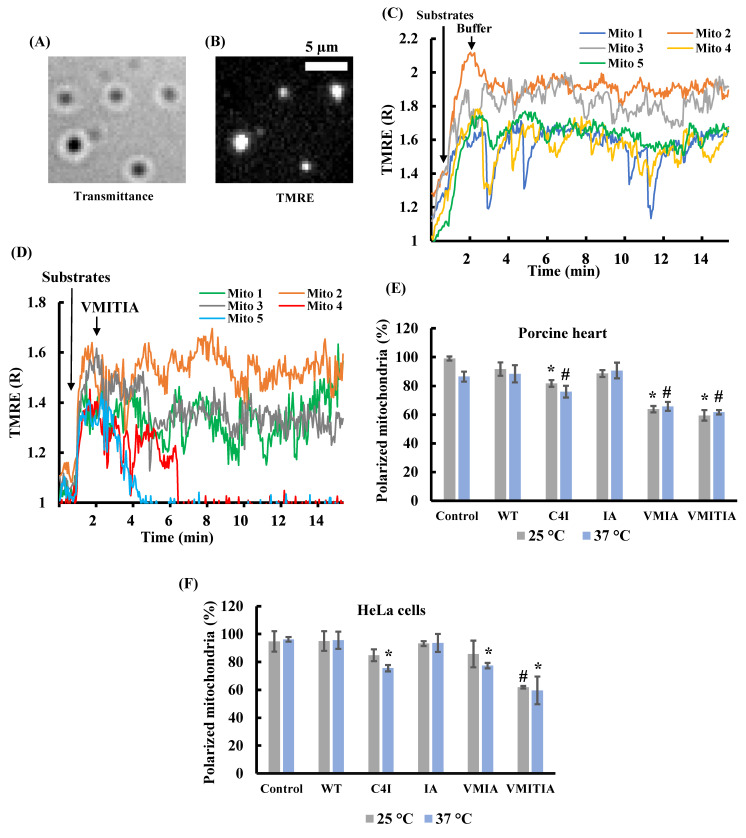
Effects of DENV3-ED3 variants on mitochondrial membrane potential. (**A**,**B**) Optical images of mitochondria isolated from porcine hearts. (**A**) Brightfield and (**B**) TMRE fluorescence images of the same microscopic field. Bar, 5 µm; (**C**,**D**) time course of the normalized TMRE fluorescence (TMRE(R)) in five isolated mitochondria. At *t* = 0.5 min, 5 mM malate and 5 mM glutamate were added. At *t* = 2 min, (**C**) Tris-sucrose buffer or (**D**) 50 µM VMITIA were added; (**E**,**F**) percentage of polarized mitochondria isolated from (**E**) porcine hearts and (**F**) HeLa cells respectively, after 10 min exposure to the indicated DENV3-ED3 variant (50 µM) at 25 or 37 °C. Polarized mitochondria were defined as mitochondria with TMRE (R) > 1.30 after malate and glutamate addition. Data represent the mean ± SEM of three independent experiments. Over 50 mitochondria were analyzed per experiment. * *p* < 0.05 vs. WT at 25 °C; # *p* < 0.05 vs. WT at 37 °C for (**E**). * *p* < 0.05 vs. WT at 37 °C; # *p* < 0.05 vs. WT at 25 °C for (**F**).

Individual mitochondria adsorbed onto a cover slip were visualized as small dots with a diameter of approximately 1 µm using fluorescence microscopy (Figure 1A,B). Since inactive mitochondria were present in the isolated mitochondrial population, we selected active mitochondria with a normalized tetramethyl rhodamine ethyl ester (TMRE) fluorescence (R) of >1.3, following the addition of 5 mM malate and 5 mM glutamate. Most active mitochondria maintained their membrane potential (TMRE (R) >1.3) in the absence of the DENV3-ED3 variants (Figure 1C). When the VMITIA variant was added, some mitochondria maintained their membrane potential (Figure 1D, Mito 1–3), while in others the membrane potential was largely lost (Figure 1D, Mito 4,5). We also compared the percentage of active polarized mitochondria 10 min after DENV3-ED3 variant addition (Figure 1E,F). The percentage of polarized mitochondria isolated from both porcine myocardium and HeLa cells was reduced following the addition of C4I, VMIA, and VMITIA variants, and this trend was more apparent at 37 °C than at 25 °C. Conversely, the wild type (WT) and IA variant did not induce significant depolarization.

### 2.2. Effects of the DENV3-ED3 Variants on Mitochondrial Inner Membrane Integrity

**Figure 2:**
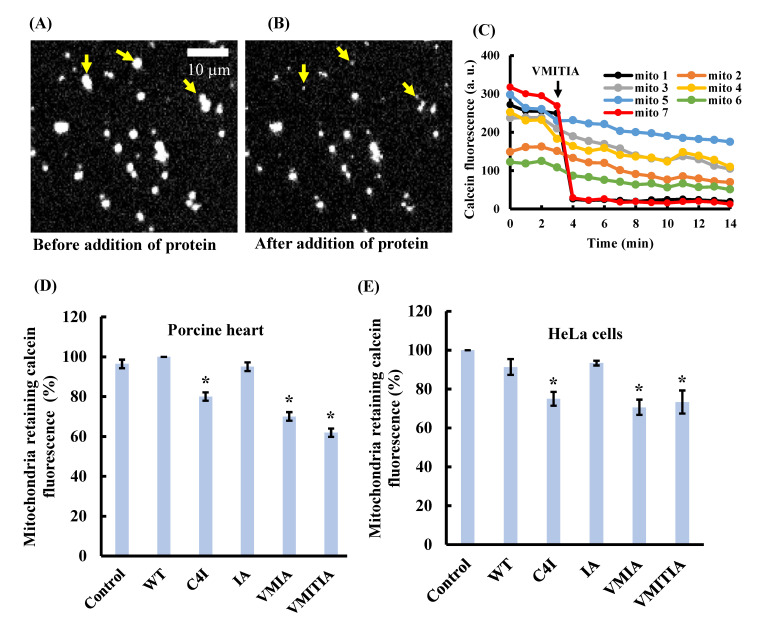
Effects of DENV3-ED3 variants on mitochondrial inner membrane integrity. (**A**,**B**) Calcein fluorescence images of mitochondria isolated from porcine hearts. Fluorescence images of the same microscopic field (**A**) before and (**B**) after the addition of 50 μM VMITIA. Arrows indicate mitochondria that released calcein after VMITIA addition. Bar, 10 µm. (**C**) time course of changes in calcein fluorescence in seven isolated mitochondria. VMITIA (50 μM) was added at *t* = 3 min. (**D**,**E**) Percentage of mitochondria that retained calcein fluorescence after 10 min exposure to the indicated DENV3-ED3 variant (50 µM) at 37 °C. Control mitochondria were treated with Tris sucrose buffer alone; (**D**) mitochondria isolated from porcine hearts and (**E**) HeLa cells. Data represent the mean ± SEM of three independent experiments. Over 50 mitochondria were analyzed per experiment. * *p* < 0.05 vs. control.

Mitochondria with intact membranes retain calcein within their matrix; however, calcein is released when the inner membrane loses its integrity [20,21]. To further understand the mechanisms of the DENV3-ED3 variants in mitochondria, we examined their effects on inner membrane integrity by detecting the amount of calcein released from mitochondria incubated with the variants at 37 °C. Figure 2A,B show calcein fluorescence images of mitochondria before (Figure 2A) and after (Figure 2B) addition of VMITIA. We estimated a >80% decrease in calcein fluorescence after 10 min of incubation with the protein mutant as a significant release of calcein (Figure 2C). Following VMITIA addition, mitochondria 1 and 7 lost calcein fluorescence, whereas mitochondria 2–6 retained their fluorescence (Figure 2C). Similar to the membrane potential findings, C4I, VMIA, and VMITIA, but not WT or IA, induced significant calcein release from mitochondria isolated from porcine hearts and HeLa cells (Figure 2D,E). Together, these results indicate that C4I, VMIA, and VMITIA impair the integrity of the inner mitochondrial membranes, and that the impairment causes the observed loss of membrane potential.

### 2.3. Effects of the DENV3-ED3 Variants on Cytochrome C Release from Mitochondria

**Figure 3:**
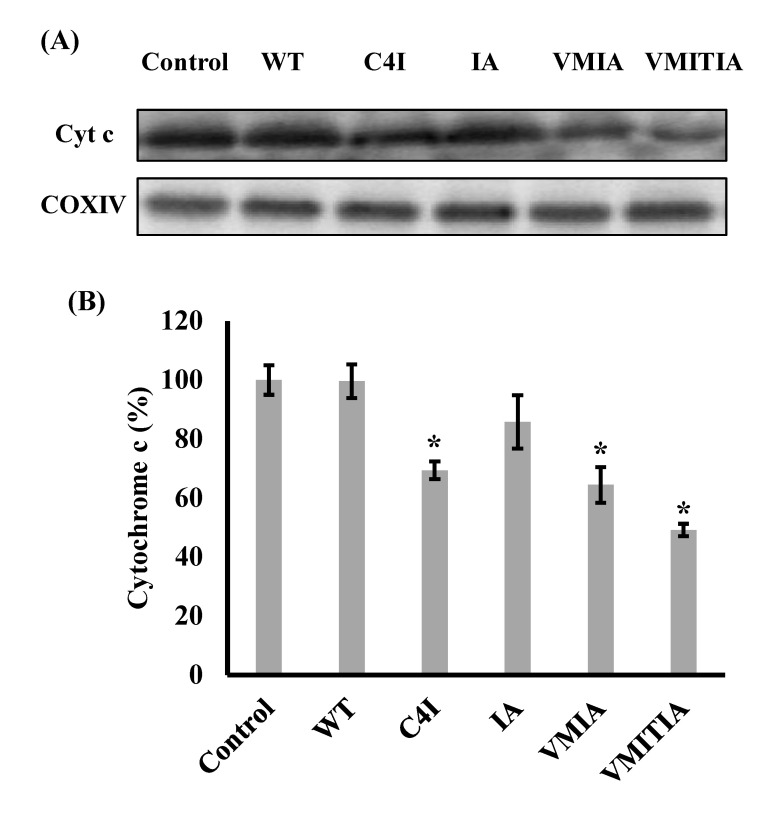
Effects of DENV3-ED3 variants on mitochondrial outer membrane integrity. Outer membrane integrity was evaluated by measuring cytochrome c release using Western blotting. Mitochondria from porcine hearts were incubated with the DENV3-ED3 variants. Control mitochondria were treated with Tris-sucrose buffer alone. (**A**) Western blot analysis of cytochrome c (Cyt.c) and cytochrome oxidase subunit IV (COXIV) inside mitochondria after incubation; (**B**) statistical analysis of the amount of cytochrome c inside mitochondria after incubation. The percentage of cytochrome c was expressed as a ratio of the band intensities for cytochrome c and COXIV. The average control values were normalized to 100%. Data represent the mean ± SEM (*n* = 3). * *p* < 0.05 vs. control.

Cytochrome c is released from the mitochondrial intermembrane space into the cytosol during the early stages of apoptosis [22] and can therefore be used to monitor mitochondrial damage. Therefore, we evaluated the release of cytochrome c from mitochondria exposed to the DENV3-ED3 mutants by performing Western blot analysis on cytochrome c and cytochrome c oxidase subunit IV (COXIV), an inner membrane protein that serves as an internal control for mitochondrial proteins. Although C4I, VMIA, and VMITIA induced a significant cytochrome c release from mitochondria, WT and IA did not, consistent with the TMRE and calcein release findings (Figure 3A,B). Together, these results indicate that C4I, VMIA, and VMITIA impair the integrity of both the outer and inner mitochondrial membranes.

### 2.4. Oligomer Size and Molten Globule-like Properties of the DENV3-ED3 Variants

**Figure 4:**
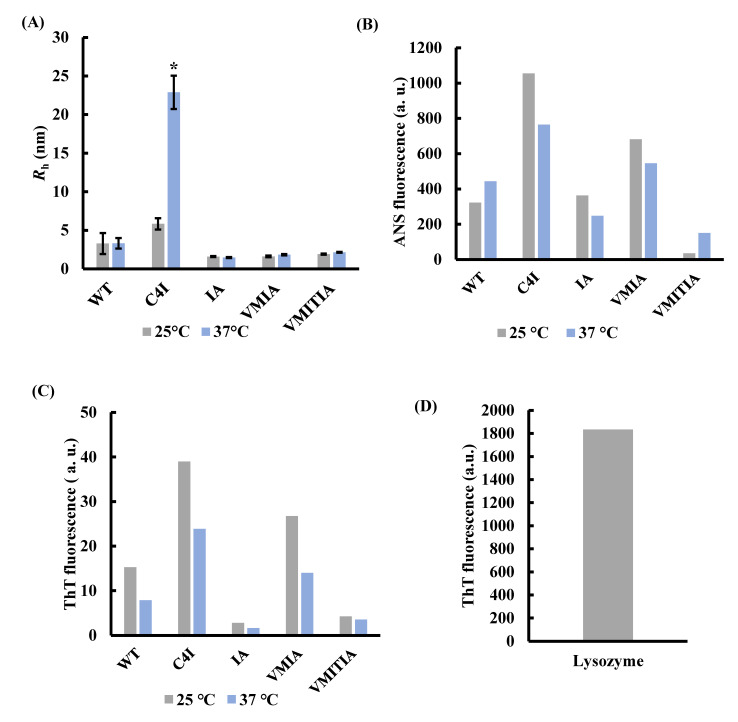
Analysis of the DENV3-ED3 variant aggregates. (**A**) Hydrodynamic radius (*R*_h_, nm) of DENV-ED3 variants determined by DLS. Data represent the mean ± SEM (*n* = 3). * *p* < 0.05 vs. WT at 37 °C. (**B**) ANS fluorescence intensity. ANS (20 µM) was added at to the DENV3-ED3 proteins (50 µM); (**C**,**D**) ThT fluorescence intensity. ThT (10 µg/mL) was added to (**C**) 50 µM DENV3-ED3 proteins and (**D**) 50 µM lysozyme.

To characterize the DENV3-ED3 variants used in this study, we first examined the size of their aggregates. Dynamic light scattering (DLS) experiments revealed that, in general, C4I formed the largest oligomers of all the DENV3-ED3 variants, while all other variants formed small oligomers (Figure 4A and Figure S2), indicating that the hydrophobic effects of the four-isoleucine tag at the C-terminus of C4I induced aggregation. Next, we examined the molten globule-like properties of the DENV3-ED3 variants using fluorescence of 8-Anilino-1-naphthalenesulfonate (ANS), which emits fluorescence upon binding to partially exposed hydrophobic patches of the monomeric DENV3-ED3 or its aggregates. ANS fluorescence assays revealed that C4I and VMIA emitted the strongest ANS fluorescence of all the mutants, suggesting that they contain large hydrophobic patches (Figure 4B and Figure S3).

The large hydrophobic region in C4I is essentially due to the four isoleucine tags at its C-terminus, whereas the hydrophobic region in VMIA is due to the steric clash between the Met310 and Ile387 side chains caused by the V310M mutation [18]. The conflicting results of the ANS and DLS assays for VMIA may be caused by the location of the hydrophobic patch, which is on the surface of the C4I protein but is relatively more internalized in VMIA. Interestingly, none of the DENV3-ED3 variants exhibited amyloidogenicity in Tris-sucrose buffer at either 25 or 37 °C, as assessed using thioflavin T (ThT) fluorescence (Figure 4C,D and Figure S4), suggesting that, under the conditions of the toxicity assays, DENV3-ED3 was either monomeric or formed amorphous aggregates, but not amyloid fibrils. Taken together, these results indicate that molten globule-like properties, aggregation size, and amyloidogenicity are not features that are common to all the mitochondria-damaging variants—C4I, VMIA, and VMITIA.

### 2.5. Secondary Structure Content and Thermal Stability of the DENV3-ED3 Variants

**Figure 5:**
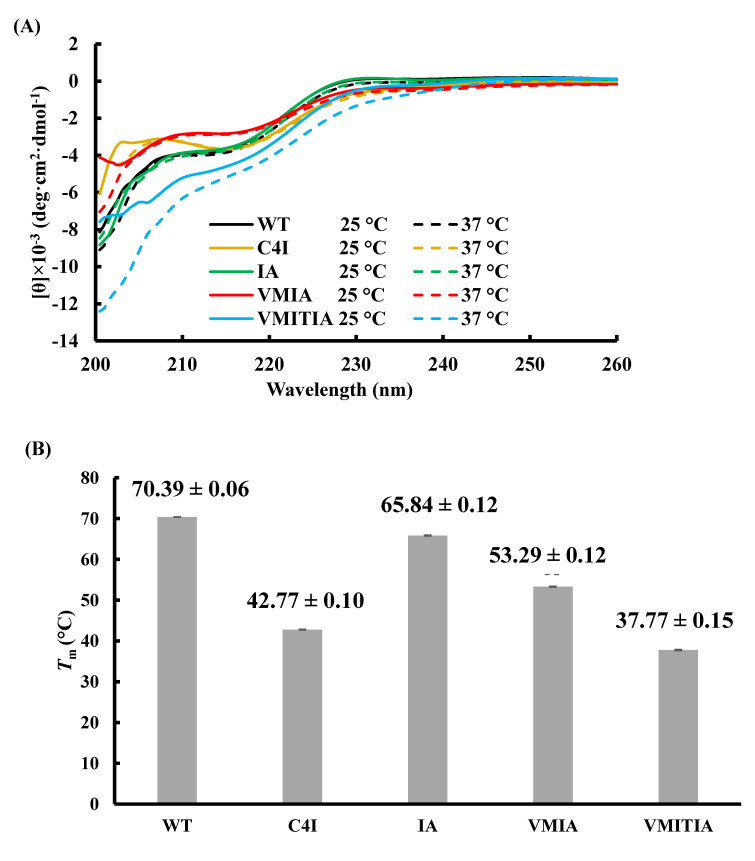
Thermal stability of the DENV3-ED3 variants. (**A**) Far-UV CD spectrum of the DENV3-ED3 variants; (**B**) melting temperature of the DENV3-ED3 variants monitored by thermal denaturation CD at 220 nm. The melting temperature (*T*_m_) was determined by fitting the thermal denaturation curve with a two-state model. Fitting errors are shown.

To further evaluate the secondary structure and thermal stability of the DENV3-ED3 variants, we performed circular dichroism (CD) analyses. As shown in Figure 5A, the far-UV CD spectra of WT and IA were almost identical but differed from those of C4I, VMIA, and VMITIA. These results suggest that the WT and IA proteins have similar secondary structures to the native protein, whereas C4I, VMIA, and VMITIA are partially unfolded at 25 °C. To examine the thermal stability of the variants, CD thermal denaturation curves were obtained by monitoring the shift in CD values at a wavelength of 220 nm with an increase in temperature (Figure S5). The melting temperature (*T*_m_) was calculated using a two-state model (N–D) (Figure 5B). Although VMITIA was the most unstable variant (*T*_m_ = 37.77 ± 0.15 °C), C4I (*T*_m_ = 42.77 ± 0.10 °C) and VMIA (*T*_m_ = 53.29 ± 0.12 °C) were less stable than the WT and IA proteins (Figure 5B). Interestingly, the *T*_m_ of the WT (*T*_m_ = 70.39 ± 0.06 °C) was close to that of IA (*T*_m_ = 65.84 ± 0.12 °C), suggesting that the I380A mutation did not alter the thermal stability of the WT protein. VMIA and VMITIA destabilization are thought to be caused by internal atomic clashes between the sidechains of the introduced mutations [18]. Conversely, the origin of the thermal destabilization of C4I is different, and can be assigned to the reverse hydrophobic effect created by the 4-Ile tag on the formation of the aggregates [23]. Thus, the thermally unstable C4I, VMIA, and VMITIA were toxic to mitochondria, but the thermally stable WT and IA did not damage the mitochondria.

### 2.6. Resistance of the DENV3-ED3 Variants to Degradation by Proteolytic Enzymes

**Figure 6:**
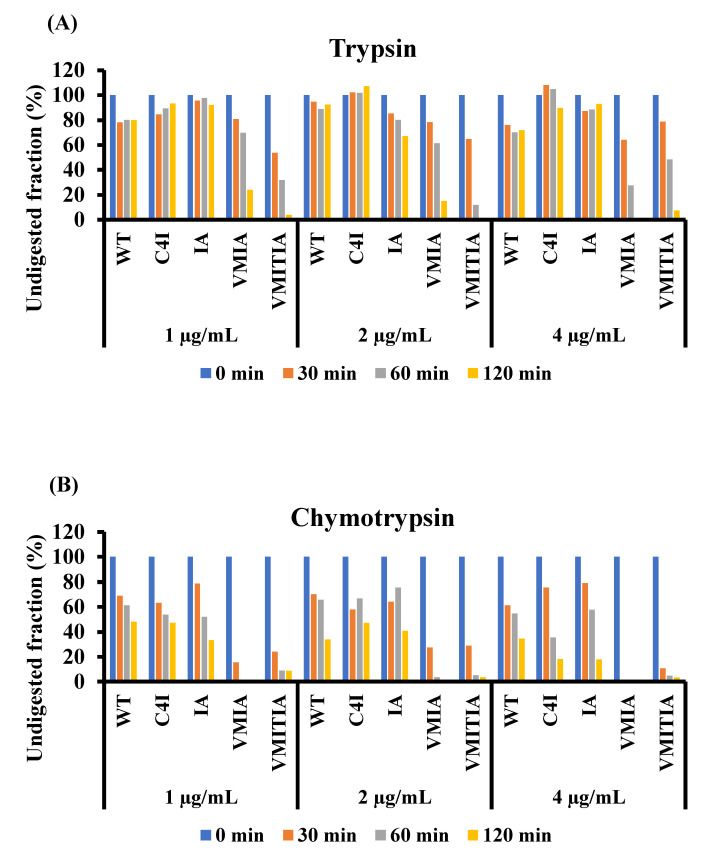
Resistance of the DENV3-ED3 variants to proteolysis. (**A**,**B**) Undigested fractions of the DENV3-ED3 variants subjected to proteolytic digestion by (**A**) trypsin and (**B**) chymotrypsin at 37 °C. The average intensity of each band was analyzed using MetaMorph (Universal Imaging) image-processing software. The value of the undigested fraction at *t* = 0 min was normalized to 100% for each concentration.

To confirm the structural differences between the DENV3-ED3 variants, we subjected each protein to proteolytic degradation using trypsin (Figure 6A) and chymotrypsin (Figure 6B). As shown in Figure 6 and Figure S6, the WT, C4I, and IA proteins were the most resistant to proteases, suggesting that their protease-mediated cleavage sites are protected by native folding (WT and IA) or oligomer formation (C4I). Oligomerized C4I is known to unfold due to the reversed hydrophobic effect caused by the concentration of the isoleucine tags, which creates a highly hydrophobic local environment within the aggregates [23]. VMIA and VMITIA were the least resistant to proteolysis, likely due to their low thermal stability which generally enhances protein flexibility or dynamics under the experimental conditions used to measure mitochondrial toxicity [24]. These results indicate that the resistance to proteases is not a common feature of C4I, VMIA, and VMITIA variants, which are toxic to mitochondria.

## 3. Discussion

Misfolded proteins and protein aggregation have been associated with various diseases; however, the mechanisms underlying the cellular dysfunction caused by non-amyloid aggregates remain unclear. In this study, we analyzed mitochondrial toxicity by DENV3-ED3 variants and characterized their biophysical properties (Table 2). Although the mutants used in this study were chemically almost identical, they possessed different biophysical properties, such as thermal stability and aggregation, which were introduced without changing the buffer conditions and by introducing a single or few mutations. In this study, we have used isolated mitochondria and performed in vitro experiments under well-controlled conditions in order to identify the biophysical properties of proteins responsible for mitochondrial damage.

The DENV3-ED3 variants used in this study were classified into two groups according to their effects on mitochondria: group 1 included C4I, VMIA, and VMITIA, while group 2 included the WT and IA proteins. Group 1 proteins acted directly on mitochondria and caused a loss of both inner and outer mitochondrial membrane integrity, whereas group 2 proteins had no effect on the membrane potential or integrity of either mitochondrial membrane. Since the loss of inner membrane integrity prevents ATP production and the loss of outer membrane integrity causes the release of cytochrome c, leading to apoptosis, group 1 proteins are likely to cause significant cell damage. Actually, several studies have demonstrated that abnormal protein-induced apoptosis is accompanied by a decrease in mitochondrial membrane potential [25,26], and there is compelling evidence that mitochondrial dysfunction plays an important role in neurodegenerative diseases caused by protein misfolding [9].

Amyloidogenicity is thought to play a major role in proteinopathies; however, our study suggests that proteins that do not form amyloid fibrils or even amorphous aggregates may still cause mitochondrial damage. Indeed, we clearly observed that the DENV3-ED3 variants caused the release of cytochrome c, which is known to induce cytotoxicity [11]. Based on their biophysical characterization, a common feature of the proteins in group 1 was thermal instability, which suggests that these proteins were highly flexible and may facilitate their interactions with mitochondria. Indeed, VMITIA, which was the least thermally stable variant, had the highest mitochondrial toxicity and was monomeric at ambient temperature, suggesting that aggregation is not required for mitochondrial toxicity.

Our study is the first to show that a thermally unstable protein that does not form amyloid fibrils, or even amorphous aggregates, can cause mitochondrial toxicity. These findings were enabled by the isolation of highly active and intact mitochondria [27], which allowed us to directly observe the effects of mitochondria–protein interactions in vitro. We were unable to fully elucidate the molecular mechanism via which thermally unstable proteins disrupt inner and outer mitochondrial membrane integrity. In addition, since the cytosol is a highly crowded environment, thermally unstable proteins resulting from misfolding may interact with other proteins before interacting directly with mitochondria. However, our results show that thermally unstable proteins with little or no tendency to form aggregates are also toxic to mitochondria. Therefore, it is conceivable that some of these proteins may interact directly with mitochondria before forming aggregates. To elucidate these matters, future studies should aim to determine the mechanism by which thermally unstable proteins become toxic to mitochondria in vivo in order to confirm the physiological significance of our findings. Although further studies are necessary, we believe that our findings provide important novel insights into the effects of protein quality control failures on cellular homeostasis.

## 4. Materials and Methods

### 4.1. Mutant DENV3-ED3 Design, Expression, and Purification

DENV3-ED3 (WT) sequences were retrieved from UniProt (ID P27915.1; residues 574 (294) to 678 (398)) [28]. The nucleotide sequence was synthesized and cloned into the pET15b expression vector (Novagen; Darmstadt, Germany) between the NdeI and BamHI sites. All mutants were prepared as described previously [28] using QuikChange site-directed mutagenesis (Stratagene; San Diego, CA, USA). The variants were named according to the number and type of amino acids added to the C-terminus of the protein [19], as summarized in Table 1. C4I had a 4-isoleucine tag added after two glycine (G) spacers at the WT C-terminus. IA had isoleucine 380 replaced by alanine. VMIA had valine 310 and isoleucine 380 substituted by methionine and alanine, respectively. VMITIA had valine 310, isoleucine 318, and isoleucine 380 replaced by methionine, threonine, and alanine, respectively.

The proteins were overexpressed in *Escherichia coli* JM109 (DE3) pLysS as inclusion bodies and purified as reported previously [28]. Briefly, the cells were lysed by sonication, and cysteines were air-oxidized for 36 h at 30 °C in 6 M guanidine hydrochloride. His6-tagged proteins were purified using Ni-NTA (Qiagen; Düsseldorf, Germany) chromatography in the presence of 6 M guanidine hydrochloride, followed by overnight dialysis against 50 mM Tris-HCl (pH 8.0) at 4 °C. After the His6-tag had been removed by thrombin cleavage, the protein was purified by a second passage through the Ni-NTA column, followed by reverse-phase high-performance liquid chromatography (HPLC) (Shimadzu; Kyoto, Japan). The proteins were identified using matrix-assisted laser desorption/ionization-time of flight (MALDI-TOF) mass spectroscopy (Bruker; Billerica, MA, USA), lyophilized, and then stored as a powder at −30 °C.

Before each experiment, the lyophilized protein powder was dissolved in ultrapure water to form a stock solution. Sample solutions were prepared at a protein concentration of 0.6 mg/mL (50 μM) in Tris-sucrose buffer containing 10 mM Tris-HCl, 250 mM sucrose, and 1 mM ethylene glycol tetraacetic acid (pH 7.4), as confirmed using a Nanodrop spectrophotometer (Thermofisher; Waltham, MA, USA).

### 4.2. Preparation of Porcine Heart Mitochondria

Porcine hearts were obtained from a local slaughterhouse and mitochondria were isolated by differential centrifugation, as described previously [27], and placed in Tris-sucrose buffer (10 mM Tris-HCl, 250 mM sucrose, 0.5 mM EGTA, pH7.4). The mitochondria were adsorbed onto a glass-bottomed culture dish (35 mm diameter) by incubating the mitochondrial suspension (0.1 mg protein/mL) on the dish at 4 °C for 90 min [29]. Protein content was determined using a BCA protein assay with bovine serum albumin (BSA) (Sigma-Aldrich; St. Louis, MO, USA) as a standard.

### 4.3. HeLa Cell Culture and Mitochondria Isolation

HeLa cells were obtained from the RIKEN BioResource Research Center (RIKEN BRC; Saitama, Japan) and maintained in Dulbecco’s modified Eagle’s medium supplemented with 10% fetal bovine serum at 37 °C in an incubator humidified with 5% CO_2_ atmosphere. Mitochondria were isolated from HeLa cells by gentle cell membrane disruption [30] and adsorbed onto a dish using the same method described for the porcine heart mitochondria.

### 4.4. Mitochondrial TMRE Fluorescence

To examine the effects of the DENV3-ED3 variants on mitochondrial membrane potential, the adsorbed mitochondria were washed twice with Tris-sucrose buffer and stained with the potentiometric fluorescent dye, tetramethyl rhodamine ethyl ester (TMRE) (10 nM; Thermo Fisher Scientific; Waltham, MA, USA), in the same buffer for 10 min at 25 and 37 °C, respectively [27]. The glass-bottom culture dish was placed on the stage of an inverted epifluorescence microscope (IX-70; Olympus, Tokyo, Japan) with a 20× objective lens (Uapo20×/340, NA = 0.7; Olympus). TMRE was excited at a wavelength of 510–550 nm emitted from a 75 W xenon lamp. Emission > 580 nm was detected using a cooled charge-coupled device camera (MD-695, Molecular Device Japan; Tokyo, Japan). To observe time-resolved fluorescence, a series of image frames were acquired at 3 s intervals with binning of 2 × 2 pixels under computational control. The exposure time for each frame was 1 s. To avoid mitochondrial damage from illumination, the excitation light was reduced to 25% with a neutral density filter and was cut off using a mechanical shutter for the remaining 2 s. The isolated mitochondria were observed at 25 or 37 °C.

The fluorescence intensity of each mitochondrion was averaged over an area of 0.6 μm^2^ using image-processing software (MetaMorph; Universal Imaging; Downingtown, PA, USA). The background fluorescence intensity (TMRE in buffer) was measured in the same field at a position where fluorescence intensity was not affected by mitochondrial TMRE. Mitochondrial TMRE fluorescence was normalized against background fluorescence to obtain the fluorescence intensity dependent on membrane potential (TMRE (R)) [31].

### 4.5. Measurement of Intramitochondrial Calcein Release

Inner mitochondrial membrane integrity was assessed by observing calcein fluorescence in the mitochondrial matrix. Briefly, mitochondria isolated from porcine hearts or HeLa cells were adsorbed on glass base dishes, incubated with 1 µM calcein-AM in Tris-sucrose buffer for 30 min at 25 °C, and then washed three times with Tris-sucrose buffer at 37 °C and kept in Tris-sucrose buffer at 37 °C. Mitochondrial calcein fluorescence was observed using the inverted epifluorescence microscope described above at 37 °C with an excitation wavelength of 470–490 nm and emission wavelength of 515–550 nm [21]. Just before observation, 5 mM malate and 5 mM glutamate were added to the mitochondria.

### 4.6. Western Blot Analysis

The suspension of mitochondria (0.6 mg/mL) isolated from porcine hearts was incubated with each protein variant (50 µM) in Tris-sucrose buffer containing 5 mM malate and 5 mM glutamate at 37 °C for 10 min and then centrifuged at 5000 rpm for 10 min at 4 °C. Once the supernatant had been discarded, the pellets were resuspended in SDS-polyacrylamide gel electrophoresis (SDS-PAGE) sample buffer (0.1 M Tris-HCl, 20% glycerol, 1 mM dithiothreitol, 4% sodium dodecyl sulfate (SDS), and 0.004% bromophenol blue, pH 6.8) and denatured at 98 °C for 10 min. Equal amounts of protein (60 µg) were then placed on each lane of a 12% SDS-polyacrylamide gel, separated using SDS-PAGE, and blotted onto nitrocellulose membranes (0.45 µm, BIO-RAD; Hercules, CA, USA). The membranes were incubated with anti-cytochrome c (Proteintech Japan; Tokyo, Japan) and anti-cytochrome c oxidase subunit IV (Proteintech Japan; Tokyo, Japan) antibodies diluted 1:5000 in phosphate-buffered saline with Tween 20 (PBST) (137 mM NaCl, 2.7 mM KCl, 8 mM Na_2_HPO_4_, 1.5 mM KH_2_PO_4_, 0.1% (*w*/*v*) Tween 20) for 1 h at 25 °C with gentle agitation. After washing with PBST, the membranes were incubated with horseradish peroxidase-coupled anti-rabbit IgG (1:10000 dilution) (Santa Cruz Biotechnology; Santa Cruz, CA, USA) for 1 h at 25 °C with gentle agitation. Specific proteins were identified using a Typhoon 8600 molecular dynamics system (BioSurplus; San Diego, CA, USA) [32].

### 4.7. DLS Assays

Prior to the experiments, stock protein solutions were centrifuged at 10,000 rpm and 4 °C for 10 min to remove precipitates and equilibrated to the measurement temperature for 10 min. Oligomer size was assessed using dynamic light scattering (DLS) with a Malvern Zetasizer Nano-S system (Malvernpanalytical; Malvern, UK) at a protein concentration of 50 μM in Tris-sucrose buffer at 25 and 37 °C, respectively. Protein samples (100 μL) were measured at least three times using disposable plastic cuvettes and the hydrodynamic radius (*R*_h_) was calculated from size–number plots using the Stokes–Einstein equation.

### 4.8. ANS and ThT Assays

1-anilino-8-naphthalene sulfonate (ANS) and thioflavin T (ThT) fluorescence were measured using an FP-8500 spectrofluorometer (JASCO; Tokyo, Japan). Prior to the experiments, stock protein solutions were centrifuged at 10,000 rpm at 4 °C for 10 min to remove precipitates and equilibrated to the measurement temperature for 10 min. The molten globule-like properties of the proteins were assessed by mixing 20 μM ANS with 50 μM protein in Tris-sucrose buffer at 25 and 37 °C, respectively. ANS fluorescence was measured at an excitation wavelength of 380 nm, and an emission spectrum of 400–650 nm was monitored. The fluorescence intensity was calculated at an emission wavelength of 470 nm.

To examine amyloid fibril formation, the protein variants were mixed at 50 μM with 10 µg/mL ThT in Tris-sucrose buffer at 25 and 37 °C. As a positive control, 690 μM chicken egg white lysozyme was incubated in 10 mM glycine buffer at pH 2 containing 0.2% sodium azide for 130 h at 57 °C [33]. Subsequently, the lysozyme solution was diluted to 50 μM in the same buffer, and mixed with 10 μg/mL ThT. ThT fluorescence intensity was measured at excitation and emission wavelengths of 440 and 480 nm, respectively. To obtain the emission spectrum, the fluorescence between 460 and 640 nm was measured upon excitation at 440 nm.

### 4.9. Far-UV CD Assays

All circular dichroism (CD) measurements were performed using a JASCO-820 spectropolarimeter (JASCO). CD spectra were obtained using CD values in the wavelength range of 200–260 nm in a quartz cuvette with a 1 mm optical path length. CD thermal denaturation curves were obtained using CD values at a wavelength of 220 nm and a scan rate of +1 °C/min in a quartz cuvette with a 1 cm optical path length. Prior to the experiments, stock protein solutions were centrifuged at 10,000 rpm at 4 °C for 10 min to remove precipitates and equilibrated at the measurement temperature for 10 min. Thermal denaturation curves were obtained at a protein concentration of 10 μM to avoid thermal aggregation at high temperatures. The melting temperature (*T*_m_) was determined by fitting the thermal denaturation curve to a two-state model using Origin 2020b (OriginLab; Northampton, MA, USA), as reported previously [15,34].

### 4.10. Proteolysis Assay

Limited proteolysis was performed using two proteases, trypsin and chymotrypsin (Nacalai Tesque; Kyoto, Japan) in phosphate-buffered saline (PBS) (pH 7.4). The protein variants were dissolved at 0.3 mg/mL (25 μM), centrifuged at 20,000× *g* at 4 °C for 10 min, and incubated with different final concentrations of trypsin and chymotrypsin (1, 2, and 4 μg/mL) for 0, 30, 60, and 120 min, respectively, at 37 °C. Approximately 5 μL of the reaction mixture was sampled after the incubation period and proteolysis was stopped by heating the sample in a loading buffer supplemented with β-mercaptoethanol at 95 °C for 2 min. Proteolysis was monitored using SDS-PAGE, and the average intensity of each band was analyzed using MetaMorph image-processing software.

### 4.11. Statistical Analysis

Data obtained using mitochondria prepared from porcine hearts and HeLa cells were expressed as mean ± standard error of the mean (SEM) of at least three independent samples. Data were analyzed using a two-tailed analysis of variance (ANOVA) followed by the Student–Newman–Keuls test. Differences were considered statistically significant at *p* < 0.05.

## Figures and Tables

**Table 1:** DENV3-ED3 variants and their definitions.

Type	Variants	Short Name	Description of the Variants
Wild type	3ED3	WT	Domain 3 of the envelope protein of the DENV from serotype 3 (DENV3)
Tag	3ED3-C4I	C4I	4-Isoleucine tag added after two
			Glycine-spacer at the C-terminus of 3ED3
Mutation	3ED3-I380A	IA	Isoleucine 380 was replaced by Alanine in 3ED3
	3ED3-V310M I380A	VMIA	Valine 310 was replaced by Methionine
			and Isoleucine 380 was replaced by Alanine in 3ED3
	3ED3-V310M I318T I380A	VMITIA	Valine 310 was replaced by Methionine,
			Isoleucine 318 was replaced by Threonine
			and Isoleucine 380 was replaced by Alanine in 3ED3

**Table 2:** Summary of the presented results.

Protein	MMP	IMM	OMM	Oligomer Sizes	Fiber (F) or, Amorphous (A)	Molten Globule-like Properties	Thermal Stability	Protein Digestion
WT	N	N	N	S	A	Lo	St	St
C4I	D	D	D	L	A	H	U	St
IA	N	N	N	S	A	Lo	St	St
VMIA	D	D	D	S	A	H	U	U
VMITIA	D	D	D	S	A	Lo	U	U

MMP, mitochondrial membrane potential; IMM, inner mitochondrial membrane; OMM, outer mitochondrial membrane; N, not affected; D, disrupted; S, small; L, large; Lo, low; H, high; St, stable; U, unstable.

## Data Availability

All the data generated in this research will be available upon reasonable request to corresponding author.

## Supplementary Materials

The following supporting information can be downloaded at: https://www.mdpi.com/article/10.3390/ijms23179881/s1.
