# Supplementary material for: Direct Analysis of Mitochondrial Damage Caused by Misfolded/Destabilized Proteins
Source: Int J Mol Sci. 2022 Aug 31;23(17):9881. doi: 10.3390/ijms23179881 (PMC9456338; doi:10.3390/ijms23179881)
Supplement: Supplementary file 1 [file ijms-23-09881-s001.zip › ijms-1870469-supplementary.pdf]

Supplemental Figures

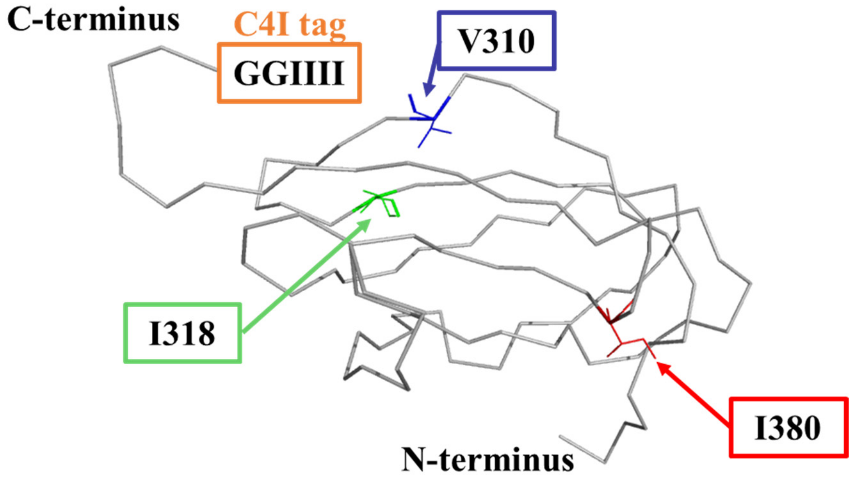

Figure S1. Related to Table1. 3D structure of the DENV3-ED3 (WT) from UniProt (ID P27915.1; residues 574 (294) to 678 (398) (PDB ID: 3VTT) in a Ribbon diagram (Elahi et al., 2013).

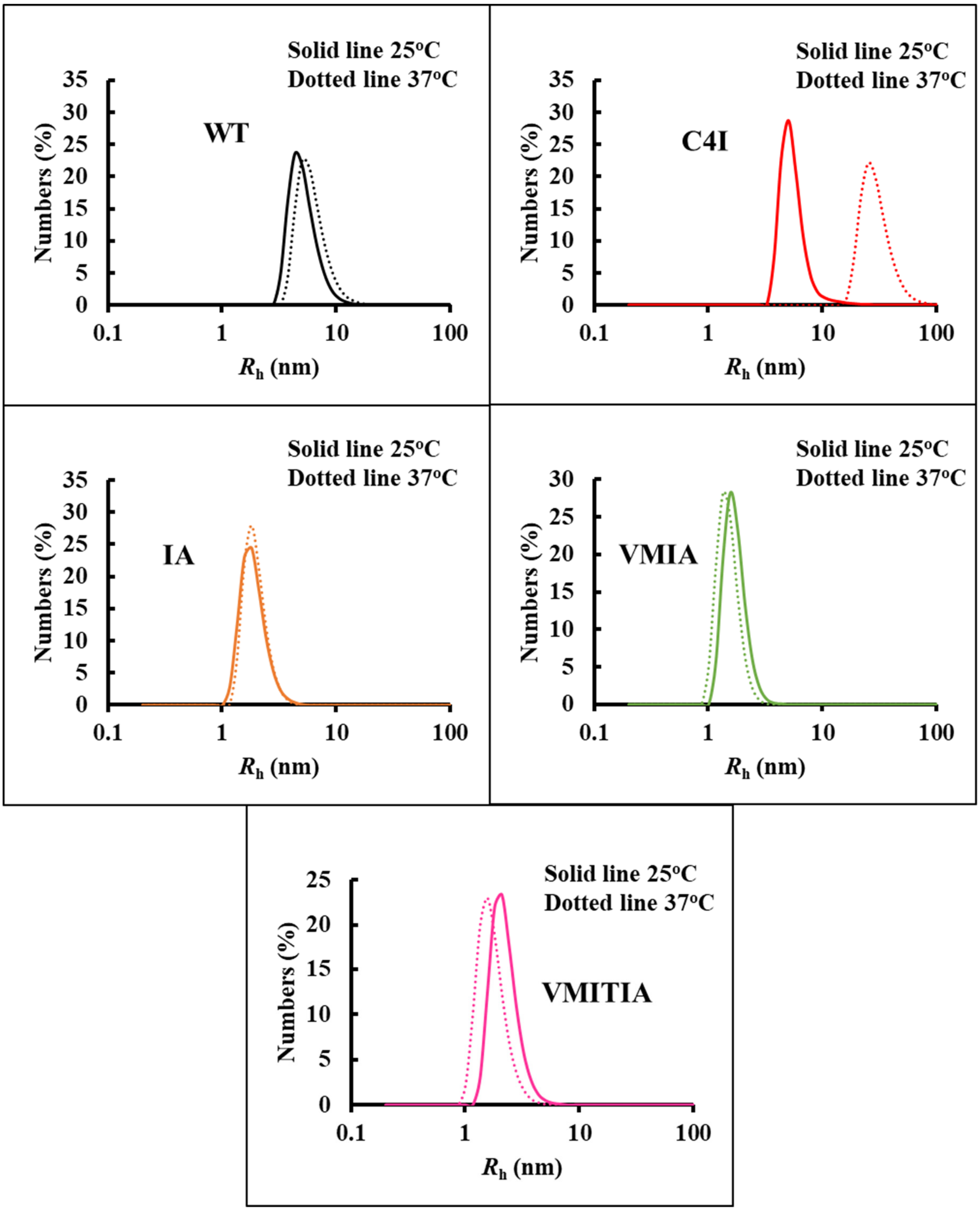

Figure S2. Related to Figure 4A. DLS spectra of the DENV3-ED3 variants were measured at 25 and 37 °C.

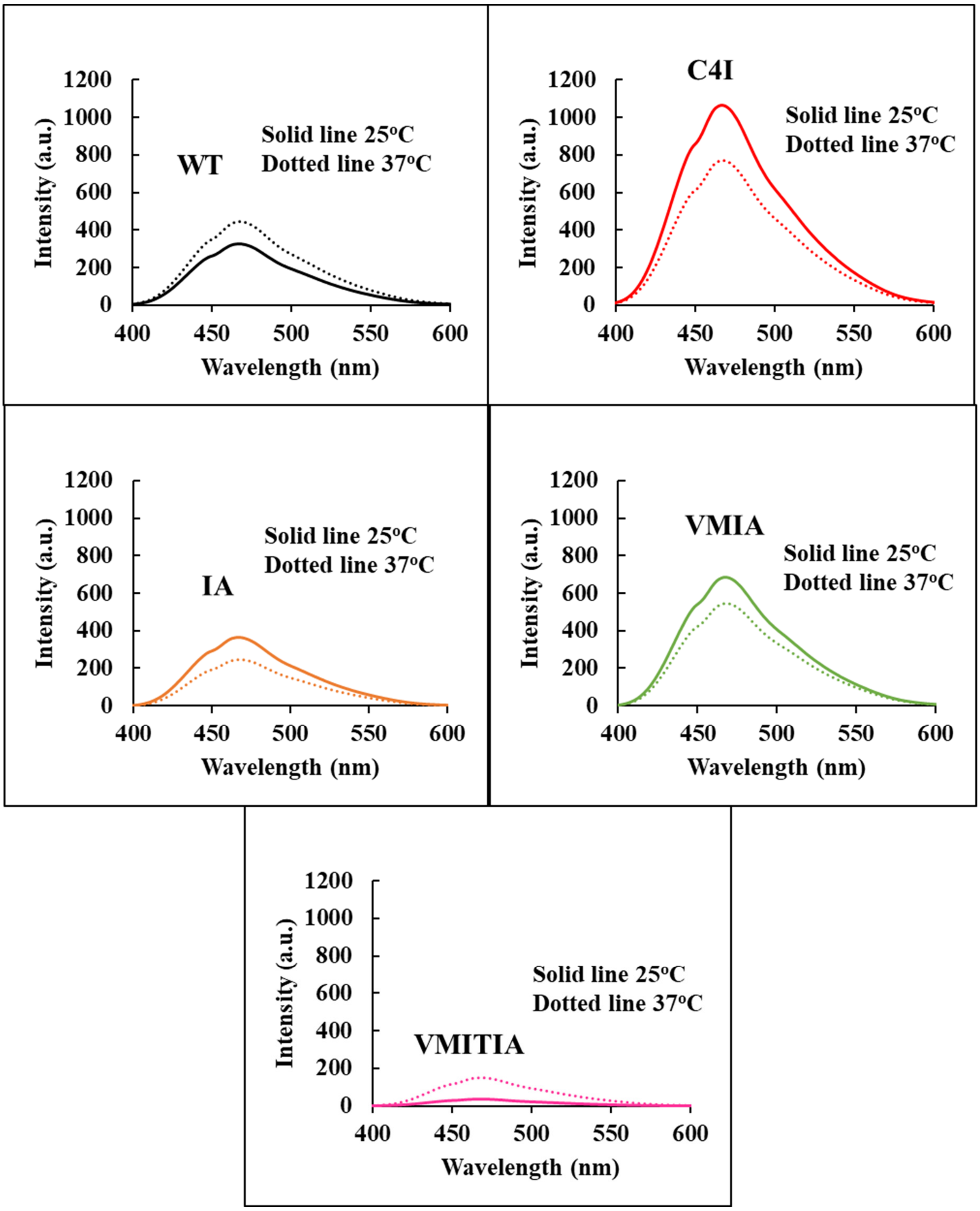

Figure S3. Related to Figure 4B. ANS fluorescence spectra of the DENV3-ED3 variants. The exposed hydrophobic regions in the DENV3-ED3 variants were assessed using ANS fluorescence at 25 and 37 °C.

(A)

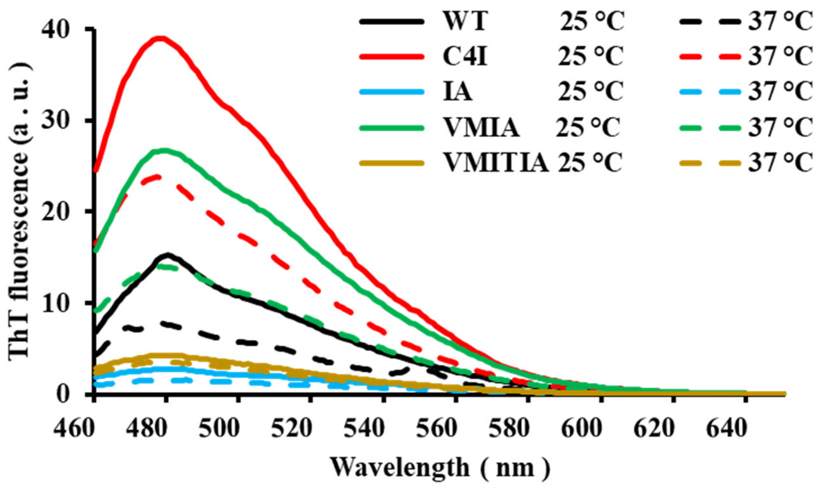

(B)

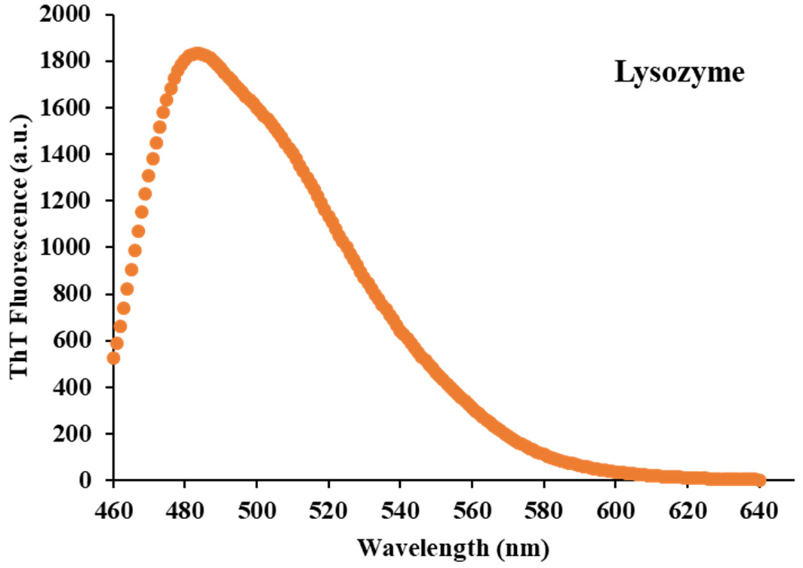

Figure S4. Related to Figure 4C,D. The amyloidogenicity of the DENV3-ED3 variants and lysozyme were measured using ThT fluorescence. (A) Related to Figure 4C. ThT fluorescence spectra of the DENV3-ED3 variants. (B) Related to Fogure 4D. ThT fluorescence spectra of lysozyme.

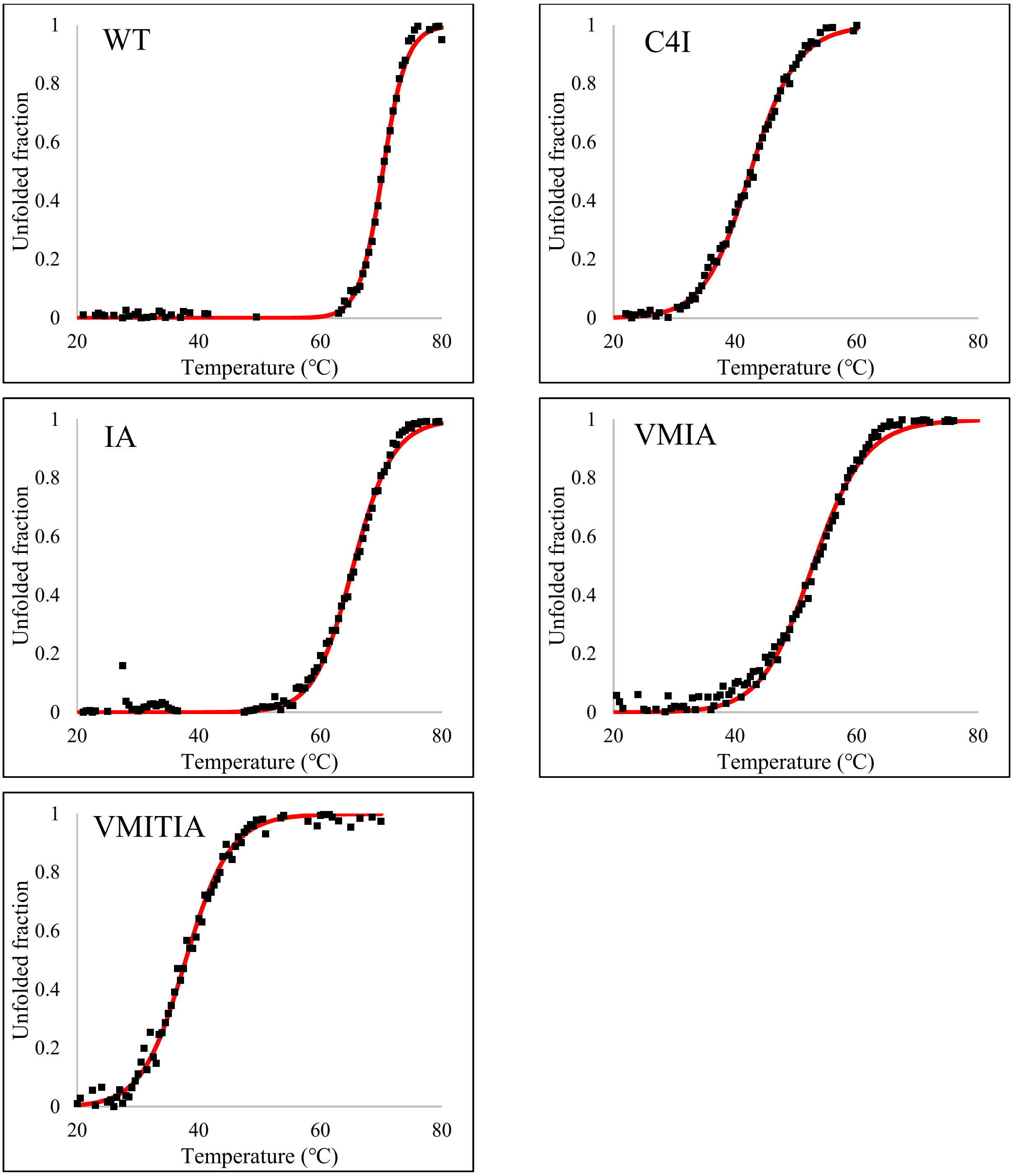

Figure S5. Related to Figure 5B. Thermal stability of the DENV3-ED3 variants measured by CD. Black dots represent raw data. Solid red lines represent fitted data. CD thermal denaturation curves were fitted with two-state thermal denaturation N↔D and converted to an unfolded fraction using the following equations: $\alpha = \frac{[N]}{([N]+[U])}$ and $\alpha = \frac{(\theta_t - \theta_U)}{(\theta_N - \theta_U)}$, where α is the fraction folded at any temperature, θ_t is the observed ellipticity at any temperature, θ_N is the ellipticity of the natively folded form, and θ_U is the ellipticity of the unfolded form.

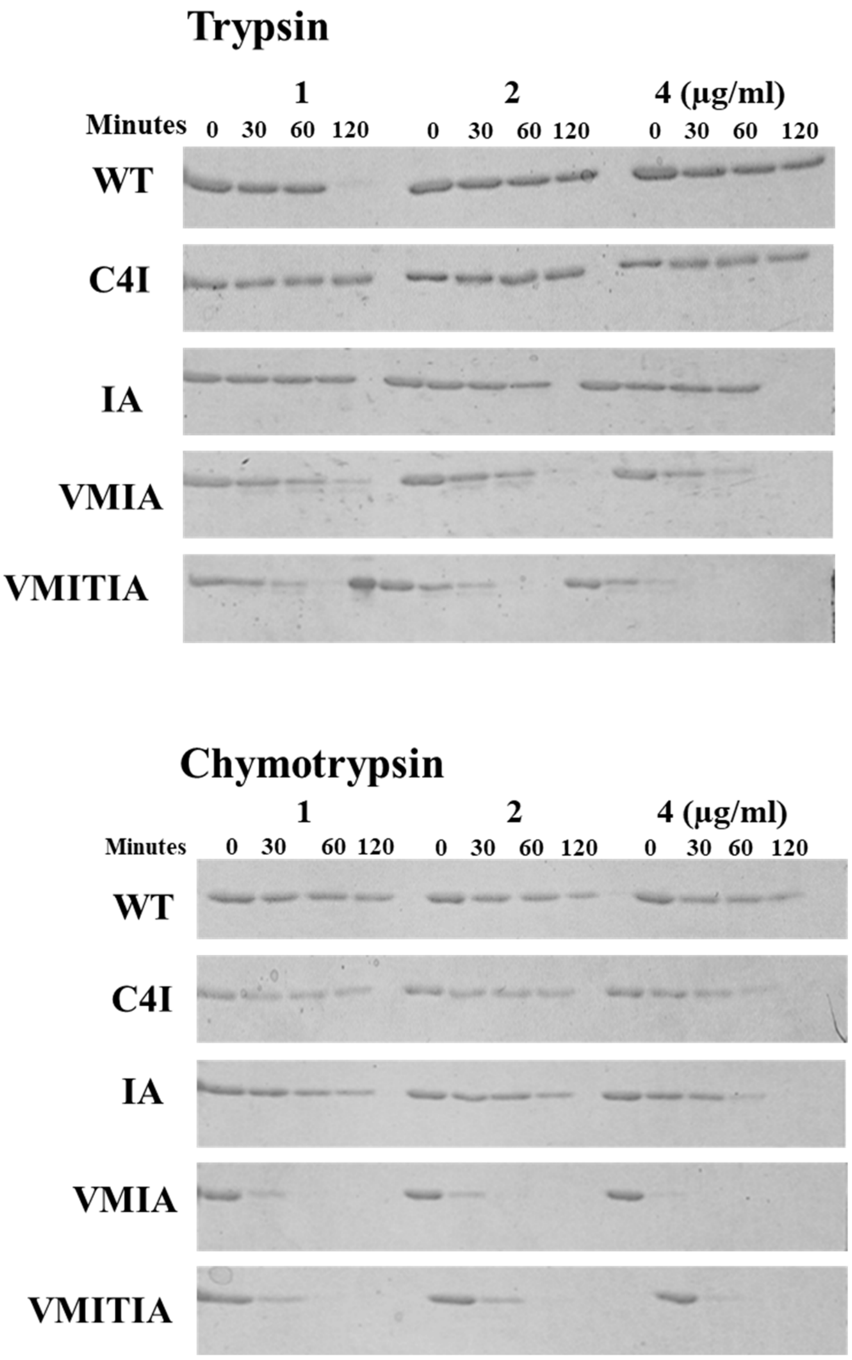

Figure S6. Related to Figure 6. Limited proteolysis of the DENV3-ED3 variants by trypsin and chymotrypsin. Lanes 1–4 on SDS-PAGE image represent 0, 30, 60, and 120 min digestion, respectively. Limited proteolysis occurred in 1× PBS at pH 7.4.
